# The Effect of Gel-Type Contributions in Lime-Sand Bricks, Alkali-Activated Slags and CEMI/CEMIII Pastes: Implications for Next Generation Concretes

**DOI:** 10.3390/gels8010009

**Published:** 2021-12-23

**Authors:** Claus Henning Rüscher, Ludger Lohaus, Fongjan Jirasit, Hervé Kouamo Tchakouté

**Affiliations:** 1 Institut Für Mineralogie, Leibniz Universität Hannover—LUH, D-30167 Hannover, Germany; 2 Institut Für Baustoffe, Leibniz Universität Hannover—LUH, D-30167 Hannover, Germany; lohaus@baustoff.uni-hannover.de; 3 Department of Civil and Environmental Engineering, Rajamangala University of Technology Lanna—RMUTL, Chiang Mai 50300, Thailand; jirasit@rmutl.ac.th; 4 Laboratory of Analytical Chemistry, Department of Inorganic Chemistry, Faculty of Science, University of Yaoundé I, Yaounde P.O. Box 812, Cameroon; htchak@yahoo.fr

**Keywords:** geopolymer-gel, lime-sand-brick, alkali-activated materials, concretes

## Abstract

Lime-sand bricks of different ages were investigated using IR-spectroscopy, thermogravimetry, and X-ray diffraction/scattering. After subtraction of the dominant quartz contribution (80%), the IR spectra show the absorption peaks of the hydrothermally formed binder phases. The spectra also show the alteration of the binder during ageing under atmospheric conditions by the influence of CO_2_ forming carbonate and a condensed SiO_2_-gel (secondary gel). The alteration could also be proven in X-ray pattern, obtaining a separation between crystalline CSH and amorphous contributions in the freshly produced lime-sand brick, too. Here, the formation of CSH_amorph_ could be understood as a precursor state (primary gel) to the crystallization of CSH phases. X-ray patterns of aged bodies of alkali-silicate solution activated slags (AAS), CEM-I/CEM-III pastes, and CEM-I concrete indicate that in all cases a similar amorphous CSH-type phase (CSH_amorph_) was formed, which is responsible for the hardening properties as the glue. The main X-ray peak of CSH_amorph_ obtained using CuK_α_-radiation with a usual diffractometer is observed between 24° and 35° 2 Theta with maximum at about 29° 2 Theta, whereas it appears much more broadly distributed between 15° and 35° 2 Theta with maximum between 26° and 28° 2 Theta for a geopolymer body prepared using the reaction of alkali-silicate solution and metakaolin (AAMK). This is due to the network formed by siloxo and sialate units in the case of AAMK, given that any crystallization can be ruled out. The origin of increasing mechanical strength during the ageing of AAS mortars must be due to further crosslinking of the preformed siloxo chains. Thermal treatment up to 800 °C leads to a complete loss of any mechanical strength of the CEM pastes due to the destruction of crystalline CSH-phases, whereas geopolymer bodies maintain their strength. Implications for next generation concrete include that cement clinker could be completely replaced by using a using alkali silcate solution technology for gel formation.

## 1. Introduction

Geopolymers in the definition of Davidovits are condensed non-periodic networks composed of poly-sialates (–Si–O–Al)_n_ and poly-siloxo (Si–O–Si)_n_ building units with a complete adaption of predominantly K and Na cations to [AlO_4_]-polyeder for charge neutrality [1]. Mixing sodium or potassium silicate solution (water glass) and calcined kaolinite (metakaolin) in a certain ratio reveals the geopolymer prototype body. The process of hardening is denoted as geopolymerisation, i.e., the crosslinking of preformed polysiloxo units from the waterglass. Meanwhile, it may also include the formation of the polysiloxo units. The formation of the polysiloxo and polysialate units and their crosslinking, geopolymerisation, could well be seen as the gel formation or gelation process. The structure of the geopolymer body shows a close analogy to the network of glasses, however not reaching their high density. A more or less high porosity permits dehydration, rehydration, and penetration with other fluids. The structural analogy could be observed in infrared absorption spectra with the three density of states peak maxima (DOSPM I, II, III) at about 480, 800, and 1100 cm^−1^ in the pure SiO_2_ glass and in bodies of sodium waterglass reacted with rice husk ash (RHA) [2]. An assignment of the DOSPM for vitreous SiO_2_ has been given previously in conjunction with a central force [AX_4_]-networks dynamics [3]. As discussed in Ref. [2], the position of the asymmetric vibration DOSPM III of the geopolymer body prepared using RHA was shown to depend sensitively on the crosslinking of chain [SiO_4_]-units (Q^2^) via further [SiO_4_]-units during ageing. The structural analogy of geopolymer and glass could also be suggested by observing the same X-ray scattering behavior showing a characteristic, X-ray amorphous bump related to pair correlations rather than any periodicity for diffraction. A first PDF (pair distribution function) analysis was conducted on a geopolymer with a Leucite analogous composition (KAlSi_2_O_6_·5.5H_2_O) [4]. Although the mean distances between pairs are close to the defined distances in Leucite in the unit cell, the true bonding distances and angles are obviously modulated from site to site, similar to possessing structure gradients in any direction. In this sense, ideal geopolymers as well as glasses may not show any nano-crystallinity.

A better distinction between the contributions of crystalline/nanocrystalline phases and X-ray amorphous contributions could be useful for a better understanding of the main phases leading to high strength concrete and mortars. In the system of C–S–H and C–A–S–H (C=CaO, A=Al_2_O_3_, S=SiO_2_, H=H_2_O), in principle, all crystalline phases are known, identified by their crystal structure and chemical—mostly rather variable—compositions. This has been summarized in detail by Garbev [5]. CSH-phases as binding phases in solidified pastes, mortars, and concretes are mostly believed to be crystalline. For example, Grangeon et al. [6] showed, that the main binding phase in hydrated Portland cement pastes with typical ratios of Ca/Si between 0.6 and 1.7 are structurally fully compatible with nanocrystalline and turbostratic tobermorite. In particular, the variations in diffraction peak intensities and peak width were shown in theoretical diffraction patterns for ordered and disordered tobermorite and jennite, for crystallites sizes 20, 10, and 3 nm. However, a question could still be related to cases where, besides the contribution of crystalline CSH phase, some X-ray scattering is present and what happens to the mechanical properties of the bulk material if this contribution becomes dominant. This is the purpose of the present contribution, to throw some more light on such examples. The usefulness of X-ray methods to quantify amorphous supplementary cementitious materials in anhydrous and hydrated blended cements is developed with the PONKCS (partial or no known crystal structure) program [7]. However, with the addition of slag, e.g., as in CEM-III, a clear distinction between reacted and unreacted slag could become rather difficult, which could lead to an overestimation of the unreacted slag contribution not observing the amorphous content as a binder.

The production of concretes and mortars always uses defined mixtures of cement, water, and gravel or sand. The cements are specified according to DIN and include, besides alite (C_3_S) and belite (C_2_S), certain further additions. In contrast, lime-sand bricks are produced following certain purity requirements using sand (of certain grain size) which is mixed and pressed together with a solution of portlandite into certain sizes before hydrothermally treatment. The Ca(OH)_2_-solution is freshly prepared from lime, CaO, and water. The hydrothermal treatment reveals a solution effect of parts of the quartz, followed by the nucleation and crystallisation of CSH phases, primarily tobermorite. This suggests that precipitates and crystals still coexist some time after production. Strong alteration will take place with increasing time. As is known, carbonation of the CSH-phases will take place with time from outside to inside due to the more or less high porosity of the as fabricated lime–sand bricks. Thus, here, the plan is to demonstrate these effects in a first step using IR-absorption, TG, and XRD measurements, comparing the results of old (20, 68 years) and freshly prepared lime–sand bricks. In a second step, our earlier results on geopolymers, alkali activated metakaolin (AAMK), and alkali activated slag (“Hüttensand”, AAH) will be discussed [2]. In this context, alkali activation means the effect of mixing an alkali silicate solution (water-glass) with MK or H, which results in solution and condensation side by side, but avoiding crystallization. According to Davidovits, alkali activated materials are not geopolymers in general. However, if ever condensation may lead to polymer formation rather than the crystallization of CSH or CASH phases, these materials may be called geopolymers, in general. Therefore, it may not be ruled out that geopolymers and CSH-phases could both be present side by side in certain cases. Investigations of the structure and mechanical properties of aluminosilicate geopolymer composites with Portland cement are also known [8]. We may also note the detailed understanding of the incorporation of Al in crystalline CSH phases leading to CASH phase [9], to be distinct from the non-crystalline gel of analogue compositions.

## 2. Investigations on Lime-Sand Bricks

**Figure 1:**
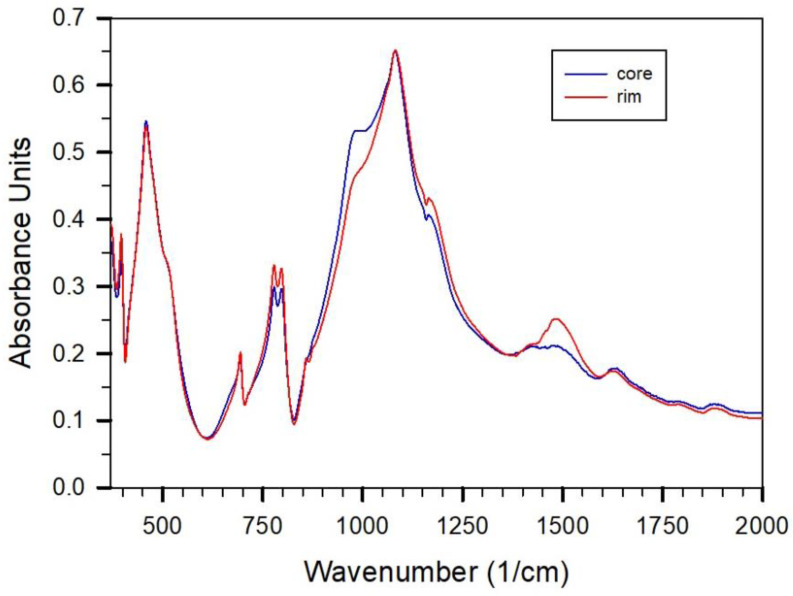
IR absorption spectra of a freshly prepared lime-sand brick probed in the rim and core showing the beginning of carbonation from outside to inside of the brick.

IR-absorption spectra on a freshly produced lime-sand brick of size 10 × 10 × 25 cm^3^ sampled close to the surface and in the middle compared to a spectrum of quartz are shown in Figure 1. It is observed that the main absorption peaks of the spectra of lime-sand brick show quartz. The quartz content can be estimated to about 80% according to the relative absorption intensities. Main differences in the spectra are observed at about 900–1000 cm^−1^ in the low wavenumber shoulder of the main absorption peak centered at 1100 cm^−1^ and between 1400 and 1500 cm^−1^. The absorption peaks between 1400 and 1500 cm^−1^ indicate the presence of calcium carbonate. A more pronounced intensity around 1480 cm^−1^ indicates the formation of aragonite in the outer zone of the lime-sandstone within days after production. An absorption with peak position at about 970 cm^−1^ can be related to the formed CSH-binder during production. Therefore, the smaller absorption intensity in this wavenumber range in the outer zone with respect to the inner zone can be related to a lower content of CSH-binder. This can be related to an increase in carbonate concentration in the outer zone. Therefore, both effects are related to the carbonation due to an uptake of CO_2_ and depletion of CSH-phase in the outer zone, formally described here as

(CaO)_n_(SiO_2_)_m_(H_2_O)_l_ + nCO_2_ => nCaCO_3_ + mSiO_2_ + lH_2_O

**Figure 2:**
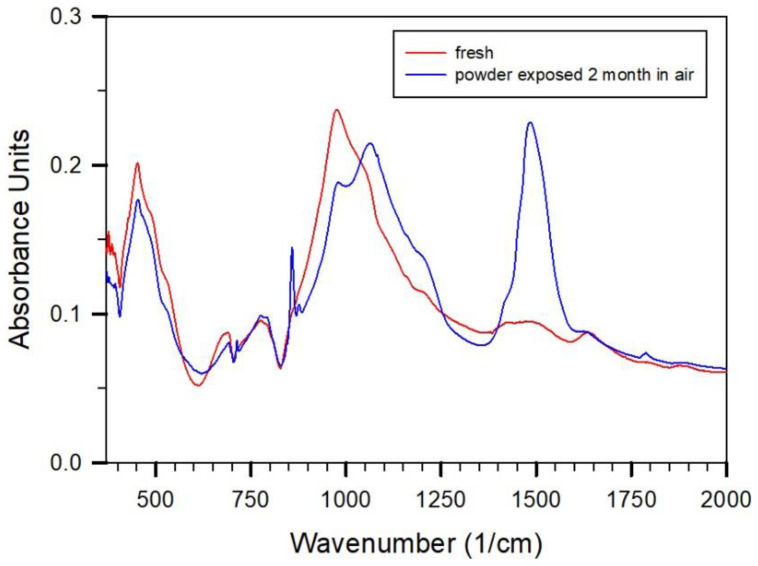
Absorption spectra of fresh lime-sand brick probed from the core (shown in Figure 1) and of powder from the core exposed for two months under open conditions. In both spectra the Qz-contribution is subtracted.

This effect can be observed more clearly after subtracting a spectrum of pure quartz. This is shown in Figure 2 for the spectrum of the sample of the core taken from Figure 1 compared to the spectrum of a powder taken from the core exposed for about two months at atmospheric conditions before pressing the KBr pellet for measurement. The pronounced absorption peak with maximum at 970 cm^−1^ is due to the hydrothermally formed CSH-phase as well as the other peaks as marked at 460 and 670 cm^−1^. It has been described by Yu et al. [10] that crystalline 1.1 nm tobermorite shows a most intense peak with maximum at 980 cm^−1^, intermediate intensity at 450 cm^−1^, and a weaker peak at 670 cm^−1^. CSH-phases which showed a single phase XRD pattern reveal, according to Yu et al. [10], rather similar IR absorption peaks to tobermorite (C/S = 0.89. With increasing C/S ratio (0.79 to 1.5), an additional peak increased in intensity around 810 cm^−1^. Yu et al. suggested a certain effect of shortening of the tobermorite specific infinite and isolated Si-O-Si-O-Si Dreierketten structure, which could be observed in small but significant variations in the main peaks. On the other hand, an additional peak around 1056 cm^−1^, a shoulder around 500 cm^−1^, and a peak between 750 and 800 cm^−1^ with an intensity similar to the Si-O-Si -chain type specific bending mode for tobermorite for a CSH-sample with C/S ratio of 0.41, was related to the coexistence of tobermorite with SiO_2_-gel as also observed in the XRD pattern as claimed by Yu et al. [10]. Following Yu et al., the spectral features related to the CSH binder in the difference spectrum of the freshly prepared lime-sand brick indicate the coexistence of amorphous SiO_2_-type gel and tobermorite-type CSH phase. The new and rather intense peaks at 860 cm^−1^ and 1490 cm^−1^ and the small one at 730 cm^−1^ show in the two-month exposed powder the formation of aragonite. The pronounced peak at about 1060 cm^−1^ indicates the formation of SiO_2_-gel from CSH-phase destruction. It contains mainly Q^3^ and Q^4^ groups similar to those described by Yu et al. for the CSH gel of C:S ratio 0.41, the formation of which was explained by polymerization. It may be suggested that the destruction of the CSH phase could involve the smallest crystals containing defects first. Whether precipitates (precursor phases) from the hydrothermal treatment in the production process of the lime-sand brick are also carbonated is hard to see. This problem could also not be solved by further XRD-investigations, as shown below.

**Figure 3:**
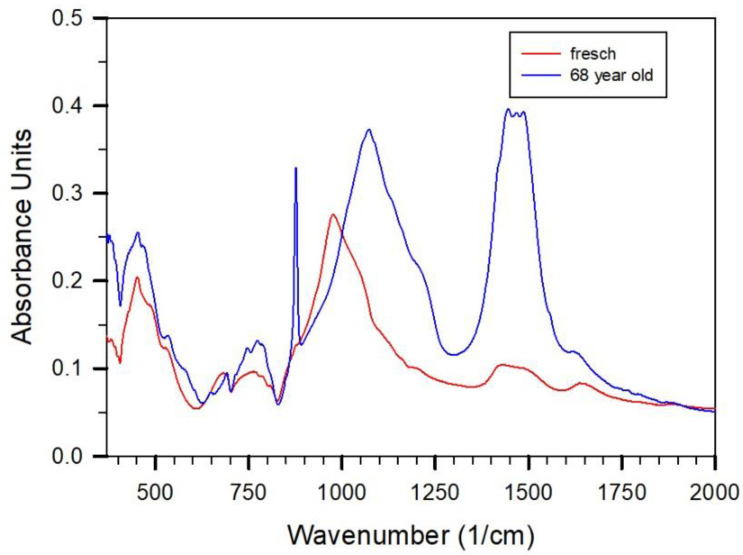
Absorption spectra of lime-sand brick probed from the core of a fresh brick (from Figure 2) and taken from a completely carbonated brick. In both spectra the Qz-contribution is subtracted.

We also observed examples of freshly produced lime-sand bricks showing just the formation of Calcite (Cc) due to carbonation. Those lime-sand bricks contained significant additions of Cc before the hydrothermal treatment in the starting mixture. Other examples which concern mainly more than 20-year-old bricks indicate the coexistence of vaterite and calcite. For example, the absorption spectra of an at least 68-year-old and exposed lime-sand brick show mainly quartz, calcite, and vaterite in the spectra with some weak variations form inside to outside of the brick. As also observed in the XRD patterns (see below), calcite appears to be a bit more intense in the outer zone compared to inside the brick. A comparison of the difference spectra (subtracting quartz) to the fresh sample from the production (Figure 3) makes evident the absence of CSH-phase as binder in the brick. In this sense, carbonation has been completed.

**Figure 4:**
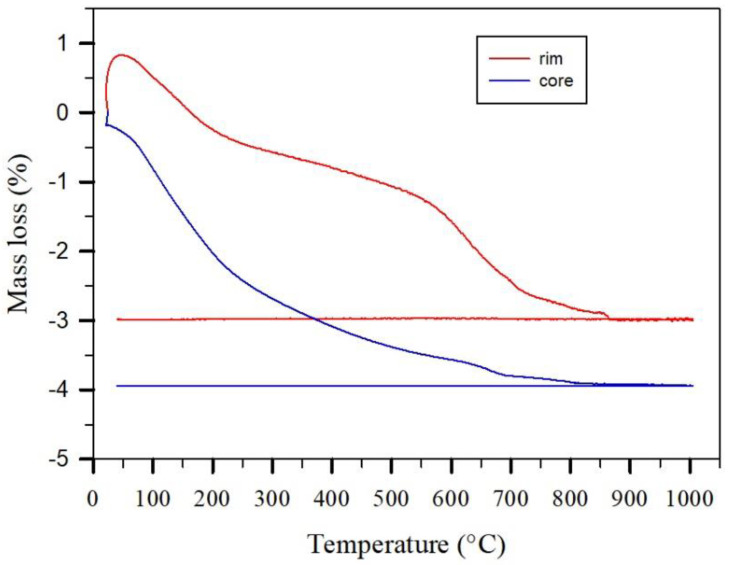
Mass loss during heating of a fresh lime sand brick probed at the core and the rim.

An easy and useful estimation of water and carbonate content is possible thermogravimetrically (Figure 4). On heating, a fresh sample from the production revealed a mass loss of about 3.7% up to 600 °C. The mass loss up to about 200 °C can be related to water in open pores and sheets related to amorphous and crystalline phases. Between 200 and 600 °C, the loss could be due to crystalline CSH phases leading to their destruction. Above 600 °C, there occurs a destruction of carbonate which amounts to a mass loss of about 0.2% due to degassing CO_2_ in the fresh lime-sand brick. The small steps at about 690 °C and 800 °C are related to the different polymorphs of calcite. Significant variations are observed in the curves of samples dependent on time of exposure to atmospheric conditions. In the present case in Figure 4, carbonation led to the formation of aragonite, which revealed a mass loss related to the destruction of carbonate of about 2% starting at about 550 °C. Small step like features are observed at about 710 and 860 °C, which again reflect the presence of different polymorphs. Clearly observed is the much smaller content of water related to crystalline CSH-phases in the sample exposed to atmospheric conditions compared to the freshly taken sample. It may be noted that the presence and content of quartz could be observed in the same run in the heatflow signals due to the α-β phase transition at 573 °C.

A strategy for the determination of the degree of carbonation was developed by Matsushita et al. [11] based on thermogravimetric investigations of hydrothermally produced concrete. These authors calculated the degree of carbonation related to the mass loss observed between 600 and 800 °C and shown by X-ray investigations and infrared absorption spectra that support carbonation as being mainly related to the destruction of tobermorite crystals. A faster carbonation of “badly” crystallized CSH-phases was suggested.

**Figure 5:**
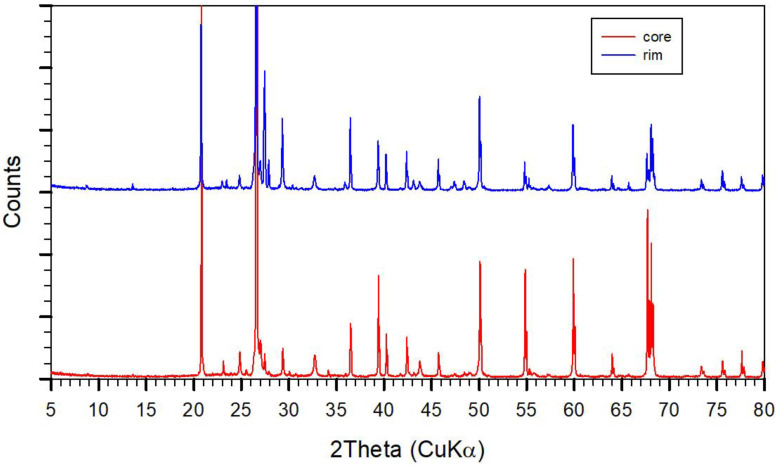
XRD-diagrams of a 68 years old lime sand brick probed at the core and rim.

**Figure 6:**
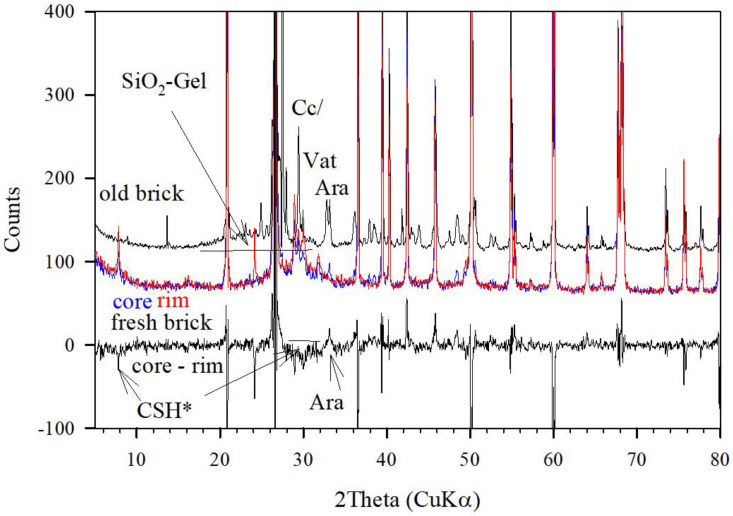
Along the ordinate enlarged XRD diagrams of a 20 year old lime-sand brick (old-brick) and a fresh lime sand brick (fresh brick), which is probed at the core and rim (plotted in blue and red onto each other. The lower graph shows the difference pattern (core-rim). Ara = Aragonite, Vat = Vaterite, Cc = Calcite. -CSH* = peaks showing the destruction of the crystalline CSH-phase in the difference pattern.

Distinctions between crystalline and amorphous contributions can be made using XRD powder investigations. However, the scattering due to amorphous contents is quite often not realized due to too short measurement times and scaling on the most intense diffraction peaks. For example, the X-ray pattern of the 68-year-old lime-sand brick obtained using a sampling from the inside and outside show mainly rather sharp diffraction peaks of Qz of high intensity due to the high content (70–80%) (Figure 5). Peaks of calcite and vaterite are also observed. The relatively higher intensity of the typically strongest calcite peak at 29.3° 2 Theta compared to the vaterite peak at 35.8° 2 Theta for the core sample support the somewhat higher content of calcite also observed in the IR spectra compared to the sample taken close to the surface (not shown). With the scaling as given in Figure 6 for a sample of an approximately 20-year-old lime-sand brick (most intense peaks of quartz are cut), a weak but significant X-ray scattering due to an amorphous contribution could be noticed as a broad contribution between 20 and 35° 2 Theta, too. After measurement and subtraction of the instrumental baseline, an integration of the total X-ray intensity (I_T_) and that of the diffraction peaks (I_C_) were carried out, the difference of which reveal the amorphous contribution (I_A_ = I_T_ − I_C_). According to this, the X-ray amorphous contribution becomes about 19% and 22% for the core and at the surface of the sample for the 68-year-old lime-sand brick shown in Figure 5. This is reliable insofar as it documents the non-crystalline part of the hydrothermally formed binder during production, which was destroyed by carbonation during ageing. A carbonate content of about 5.5% and 7% could be estimated thermogravimetrically for the inside and surface-related samples, respectively. The water content could be estimated to about 2%. All methods, i.e., XRD, IR, and TG, showed a somewhat higher content of carbonate in the outer zone compared to the interior. This could also indicate a more significant consumption of quartz in the outer zone compared to the inner zone either during production or even during ageing. Further investigations in this direction could be interesting.

The XRD pattern of a lime-sand brick aged about 20 years old in open conditions shows an almost complete carbonation, too (Figure 6). The amorphous contribution, denoted as SiO_2_-gel, can be seen here as the broad peak between 18° and 32° 2 Theta. By integration, about 20% intensity of the amorphous part (I_A_) is obtained, similar to that obtained in the 68-year-old lime-sand brick (Figure 5). The thin line closely approximates the instrumental baseline, showing that significant scattering contributions are also obtained up to 70–80° 2 Theta. However, a further quantification of this requires improved instrumental conditions, as known for PDF-analysis techniques [4], which is outside the scope here. For a further discussion of the relative crystalline and amorphous contributions, the treatment of the data as follows might be sufficient. For the 20-year-old lime-sand brick, the peaks at 34.2 and 35° 2 Theta indicate the formation of aragonite and vaterite side by side with calcite (29.3°). There are also rather weak but narrow peaks at 28.0° and 30.0° 2 Theta which show that tobermorite crystals are still present, although an adequate (001) peak at 9° 2 Theta (11A-Tobermorite) is missing. This indicates the absence of any significant sheeted ordering of crystalline extension. Thus, the crystals may show a very thin needle type shape. A comparison to the X-ray pattern of the freshly produced lime-sand brick obtains a similar broad peak of similar intensity, which is however shifted to between 22 and 34° 2 Theta with a maximum at about 28–29° 2 Theta. This broad peak may also indicate an X-ray amorphous contribution rather than being related to small CSH-crystals. On the other hand, the XRD-pattern of the freshly prepared lime-sand brick shows significant contributions of well-formed 11-A tobermorites, as deduced by the characteristic peaks at 9, 28, and 30° 2 Theta. A closer comparison of the X-ray patterns sampled in the middle and close to the surface of the freshly prepared brick proves in the difference pattern (Figure 6) that carbonation forms aragonite, as was also observed in the IR absorption spectra (Figure 1). Parallel to this, a destruction of tobermorite crystals occurs, very probably beginning most significantly from the smallest and less stable crystals, which could be identified in the difference pattern, too. During further carbonation, a complete extraction of Ca-ions, or CaO-units, occurs, leading to the crystallization of CaCO_3_ and formation of SiO_2_-gel. This may form another (secondary) glue for the lime-sand-bricks during ageing.

## 3. The X-ray Pattern of AAMK, AAH, CemI/III Pastes and High Strength CEM-I Based Concrete

**Figure 7:**
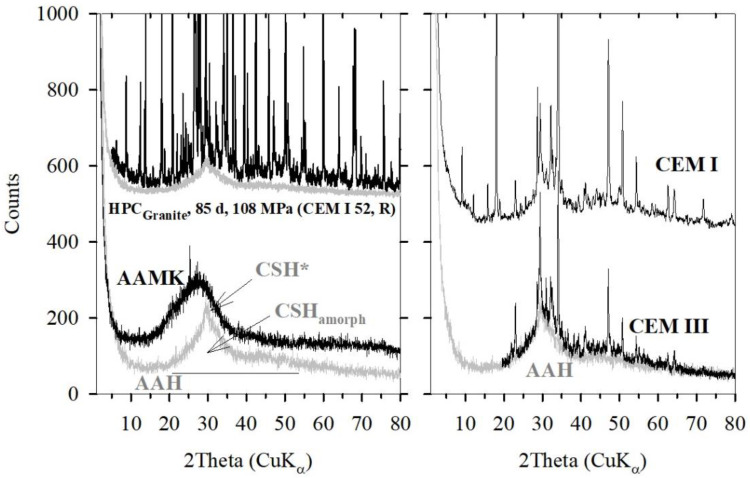
XRD-diagrams of AAMK, AAH and of a high strength concrete, HPC, (**left**) compared to those of pastes of CEMI, CEMIII (**right**). The AAH pattern is shown three times for better direct comparison to AAMK, HPC and CEMIII pattern.

X-ray patterns of alkali activated metakaolin (AAMK), alkali activated slag (AAH), CEM-I and CEM-III pastes, and a high strength CEM-I based concrete are shown in Figure 7. The X-ray pattern of AAMK is a typical example of a geopolymer body. There are no crystalline contributions disregarding spurious contents of anatase related to the otherwise very pure kaolinite source material. Whereas the “amorphous bump” of the AAMK sample is rather broadly distributed between 15 and 35° 2 Theta with the main maximum at about 25–28° 2 Theta, the X-ray pattern of an AAH body shows a distribution restricted to 20–35° 2 Theta and a weak peak with a maximum at about 29° 2 Theta. Since the AAH composition include mainly CaO and SiO_2_ and less Al_2_O_3_, the reaction with waterglass thus forms condensed SiO_2_ chains which become mainly crosslinked by siloxo-units and less by sialate units. Crosslinking the chains mainly by sialate-units may lead to additional X-ray scattering contributions, as seen for the AAMK sample. In this sense, the amorphous peak of the AAH sample, denoted as CSH_amorp_ in Figure 7, is distributed rather similarly in the same range also observed for the freshly prepared lime-sand brick (Figure 6) and for CEMI and CEM II pastes, too. The direct comparison of the X-ray pattern of alkali activated slag to a paste prepared using CEM-III and water shows rather close agreement in the amorphous contribution. This may be expected since CEM-III contains beside C_2_S and C_3_S up to 60% slag. It could be estimated that the X-ray amorphous scattering is about 55%. For the X-ray pattern of CEM-I paste, a similar X-ray amorphous contribution of about 40% is obtained, too. A similar X-ray amorphous content of about 30% is obtained for the high strength concrete.

**Figure 8:**
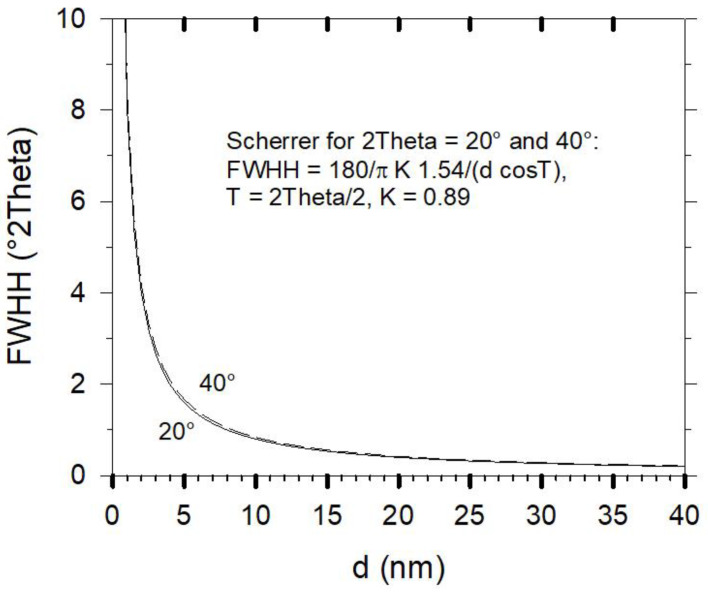
Plot of the Scherrer function for FWHH (full width at half height) of diffraction with CuKα1 at 20° and 40° 2 Theta in the range d < 20 nm.

The weak peak at 29° 2 Theta denoted by CSH* on top of the broad “amorphous bump” of the AAH sample is related to some small content of nanocrystalline CSH-phase. The FWHH of this peak is estimated to be no more than about 1° (CuK*a*_2_ subtracted). Using the Scherrer formula shown in Figure 8 would indicate a periodicity of about 10 unit cells along the chain direction of the Dreierketten in tobermorite (b = 0.73 nm). For a further decrease of their size, a breakdown of diffraction may rapidly be reached. It may also be noted that the scattering effect of AAMK and AAH samples is not only concentrated in the “amorphous bump” range, but also present in the higher 2 Theta range. As mentioned in the introduction, more advanced X-ray methods are required for a better data evaluation of these contributions.

## 4. Distinction between Crystalline and Amorphous CSH by Thermal Treatment

**Figure 9:**
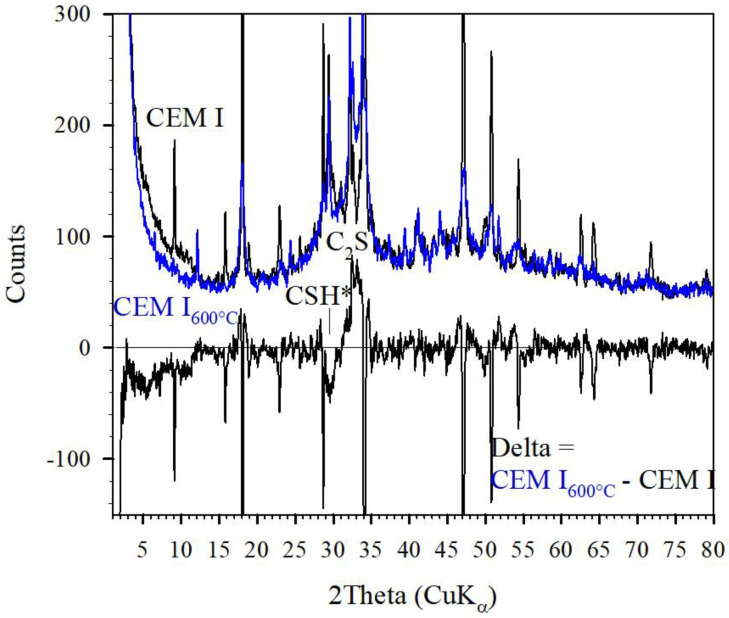
XRD diagramms of CEMI-stone sample before and after thermal treatment at 600 °C and the difference pattern as denoted.

A distinction between the amorphous contribution and the crystalline CSH-phases in cement stones is obtained using a thermal treatment of samples. A thermal treatment up to 800 °C did not weaken the compressive strength of the AAMK sample, 35–31 MPa [12]. The XRD diagram remains the same as shown in Figure 7. This is also supported by results given by [4]. CEM-pastes lost 100% of their strength when thermally treated at 800 °C. It may also be noted that the ultra-high performance concrete reveals strength variation of about +/− 20% when heated up to 800 °C as obtained in an in-situ study by Hosser et al. [13]. It is known that crystalline CSH-phases become destructed due to dehydration during heating. For comparison, the X-ray patterns of a CEM-paste before and after thermal treatment at 600 °C are shown in Figure 9 together with the difference pattern (Delta = CEM-I_600°C_ − CEM-I). It is evident that the destruction of the nano-crystalline CSH phase (CSH*) could lead to the crystallization of C_2_S. No change in the amorphous contribution is obvious. It is concluded that the loss in crystalline CSH* led to a loss in compressive strength. On the other hand, AAMK retain their strength when heated up to 800 °C where dehydration and rehydration occurs similarly to zeolite.

**Figure 10:**
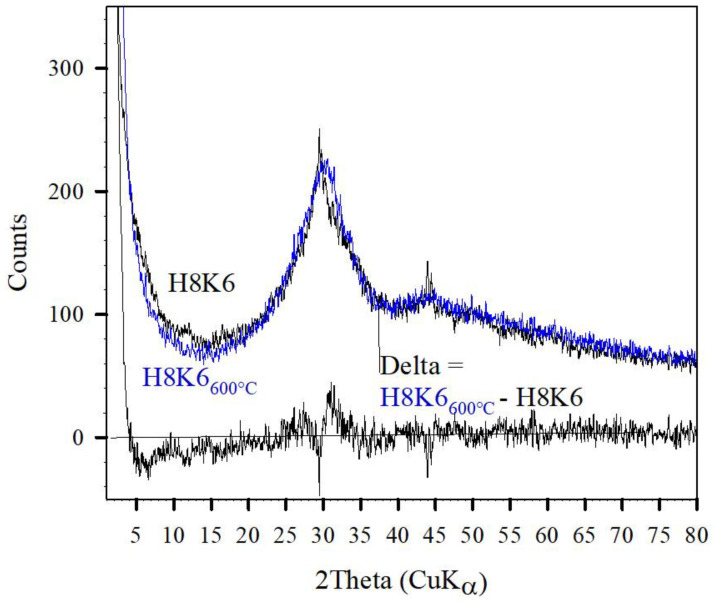
XRD diagramms of AAH sample before and after thermal treatment at 600 °C and the difference pattern as denoted.

A similar distinction between amorphous and crystalline contributions is also obtained for AAH samples. It has been argued that the XRD-pattern of AAH shown in Figure 7 was assigned mainly to CSH_amorphous_ and to only a minor content of crystalline CSH*. Conclusively, heating at 600 °C reveals the destruction of the CSH* contribution as exemplified in Figure 10 for another AAH sample before and after thermal treatment at 600 °C. This sample revealed compressive strength of about 100 and 55 MPa before and after thermal treatment at 800 °C, respectively [12].

## 5. Implications for Mechanical Strength Development

**Figure 11:**
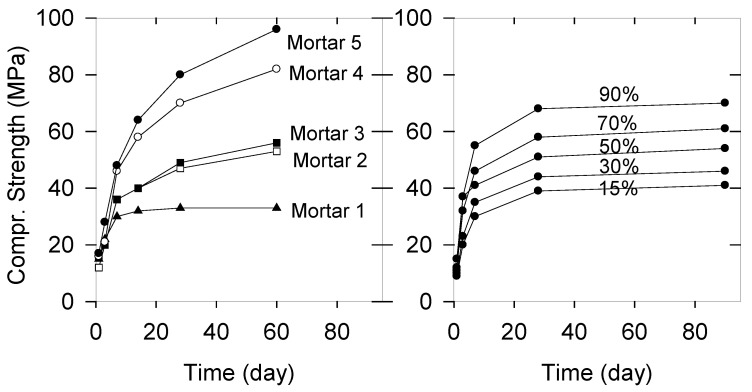
Compressive Strength data of mortars hardened using alkali activated metakaolin (Mortar 1), alkali activated slag (Mortar 5) and mixtures MK/H 10/90, 30/70, 50/50 (Mortar 2, 3, 4) (**left**, taken from Ref. [2]), and of blends (50% clinker, 30% calcined clay, 15% limestone 5% gypsum) with calcined kaolinite in portions 15, 30, 50, 70, 90% (w/c = 0.5, sand/cement = 3 (**right**, data taken from Ref. [15] as an estimated average). Mortars in Ref. [2] use WG K20/SiO_2_ 0.5 with 8 M KOH, WG/solid 0.75 and sand/cement = 3).

Questions remain concerning the contribution of the gel or geopolymer formation on the mechanical properties and the extent to which any high temperature cement production could be avoided. Compressive strength data for mortars using AAMK and AAH and alkali activated mixtures of MK/H obtained following 60 days [2] and after five years [14] were reported. Data from [2] are shown in Figure 11 (left) compared to data taken from the work of Scrivener et al. (right) [15]. These authors investigated the impact of calcined kaolinite content on mortar strength for blends of 50% clinker, 30% calcined clay, 15% limestone, and 5% gypsum (so called LC^3^-50 mixture). Scrivener et al. reported that the LC^3^-50 mixture with calcined clay, which contained only 40% calcined kaolinite, already demonstrated mechanical properties comparable to those obtained using plain Portland cement from about seven days (w/c = 0.5, sand/cement = 3). It could be shown that the mechanical strength practically increases linearly with the content of calcined kaolinite. For comparison, using alkali activated mixtures of MK/H of about 10/90, 30/70 obviously shows similar performances to those reported for 50–70% calcined clay additions with LC^3^-50 mixtures. In this respect, AAMK mortars reveal a compressive strength of about 35 MPa which also showed the same strength after five years [14]. For AAH mortars, a rather steep increase up to 65 MPa within 14 days followed by a value of 90 MPa after 60 days was measured. After five years, the compressive strength reached 140 MPa, remaining at the same value after 10 years [unpublished]. Any significant carbonation could be ruled out. We could not clarify whether an increase or decrease in the nanocrystalline contribution happened relative to the X-ray amorphous scattering. Based on the significant change in compressive strength within about five years, a significant degree of reaction can be suggested. The only source could be a certain content of unreacted slag which could be enforced by a residual alkaline solution created by water from a condensation reaction.

## 6. Conclusions

1. IR-difference spectra, i.e., after subtraction of the quartz contribution, show the characteristic absorption features of crystalline and amorphous binder in the freshly produced lime-sand brick. The peak position at 970 cm^−1^ together with the absorption peaks at 670 cm^−1^ and at 450 cm^−1^ indicate the formation of crystalline–CSH phase, tobermorite, with the characteristic Si–O–Si–O–Si Dreierkettenstruktur. Carbonation enforces its destruction and the formation of a SiO_2_-Q^3^-network (DOSPM at 1060 cm^−1^), denoted SiO_2_-gel. Thermogravimetric analysis provided an easy estimate of the carbonate content. X-ray patterns of freshly produced lime-sand bricks showed, besides quartz as the main ingredient, hydrothermally formed tobermorite crystals and some broad X-ray contribution between 25 and 35° 2 Theta, which was related to a non-crystalline CSH-type gel (primary gel) rather than nano-crystalline CSH. The X-ray patterns of fully carbonated lime-sand bricks show a new, broadly distributed amorphous contribution between 15 and 32° 2 Theta related the SiO_2_ gel (secondary gel) formed by Ca extraction from the CSH-phases.
2. Alkali activated slags (AAH) show, compared to geopolymers obtained by alkali activated metakaolin (AAMK), a narrower distribution of the “amorphous bump”, mainly between 20 to 35° 2 Theta and with maximum around 29° 2 Theta, which is related to the two different types of gel, particularly the high Ca-content structurally bound between SiO_2_-chains denoted here as CSH_amorph_. In the X-ray pattern of AAH, a rather small contribution of nano-crystalline CSH phase (CSH*) was observed, too. X-ray patterns of CEM-I paste, CEM-III paste, and CEM-I based concrete show close agreement with the X-ray pattern of AAH with the addition of crystalline contributions.
3. XRD of samples taken before and after thermal treatments could be used to discriminate between CSH* and CSH_amorph_ (here: crystalline and amorphous contents in AAH and CEM pastes)
4. Finally, it is shown that adequate mechanical strength is obtained not only by using calcined clay of a certain quality (content of metakaolin) for the partial replacement of clinker phases in cement. As an alternative, mixtures of AAH and AAMK could well be used. Thus, a technology based on “alkali-activation” for gel formation could completely substitute the requirements of using alite and belite. At this point, we may refer to some investigations and more references therein concerning alternative sources substituting slag (H) for obtaining (Ca, Na, K)-poly(sialate-siloxo) networks as binders for sustainable building constructions, e.g., [16,17], and the effect of calcium phosphate compounds on the mechanical and microstructural properties of metakaolin-based geopolymers, e.g., [18]. Other directions of recent research concerning new binders for building construction are acid-based geopolymers, including calcined laterites [19].

## 7. Methods and Samples

IR investigations were carried out using the KBr method (1 mg sample dispersed in 200 mg KBr) on an FTIR spectrometer (Vertex 80v, Bruker Optics, Karlsruhe, Germany). TG/DTA experiments were conducted using an approximately 50 mg sample in Al_2_O_3_-crucibles (Setsys evolution 1650, Setaram, Paris, France). XRD analyses were carried out in Bragg–Brentano Theta/2 Theta geometry (D4, Bruker AXS, Karlsruhe, Germany) using graphite monochromised Cu K_α_ radiation. In part 3, commercially available lime sand bricks were used: fresh from the production line, an approximately 20-year-old brick from a private source (CHR) and a 68-year old brick from the archive of ZMK eV (Hannover; Germany). Results of XRD in part 4 were obtained on a sample concrete as denoted HPC_Granite_, prepared using crushed granite and CEMI 52R. It shows compressive strength of 108 MPA tested at 85 days. Samples denoted AAMK, AAH (Figure 7), and H8K6 (Figure 9) were originally prepared as described in Ref. [2] and further in Refs. [12,14] and could be taken from our archive. The preparation of CEM I and CEM III pastes are described in Ref. [12] and could be taken from that reservoir.

## Data Availability

Data obtained as described.
